# Bringing Sectors Together in Da Nang, Vietnam: Participatory Systems Mapping

**DOI:** 10.1007/s11524-022-00650-6

**Published:** 2022-07-05

**Authors:** Thi Kinh Kieu, Karen Grattan, Bailey Goldman, Tran Thi Thuy Ha, Tran Thi Thu Thi, Amanda Pomeroy–Stevens, Damodar Bachani

**Affiliations:** 1 The University of Danang, University of Science and Education, Da Nang, Vietnam; 2 Building Healthy Cities Project, Engaging Inquiry LLC, Durham, NC USA; 3 Building Healthy Cities Project, Thrive Networks/East Meets West Foundation, Da Nang, Vietnam; 4 Building Healthy Cities Project, JSI Research & Training Institute, Inc, Arlington, VA USA; 5 Building Healthy Cities Project, John Snow India Private Limited, New Delhi, India

**Keywords:** Urban health, Systems mapping, Smart City, Da Nang

## Abstract

The USAID-funded Building Healthy Cities (BHC) work in Da Nang, Vietnam, engaged 108 multi-sector stakeholders to gather qualitative data across two workshops and three citizen town halls from 2019 to 2021. These data were synthesized with the results from BHC’s seven other activities in Da Nang to build systems maps. Contextual findings showed that multi-sector, multi-level participation and collaboration have been the key to moving the city toward their smart and livable city goals. Currently, citizen, nongovernmental organization, and private sector collaboration are low for many government sectors, which results in policy and programs that are mismatched to actual needs and therefore have less powerful impacts. When these policies and programs are implemented, they struggle to demonstrate strong benefits to these stakeholder groups, further decreasing participation. This is central to the systems map that BHC developed, and is expanded upon through additional patterns that fall within four main areas: management quality; vision and leadership; workforce capacity; and community engagement. Stakeholders found four key leverage points within this context that, if included in every action, could help overcome barriers. These leverage opportunities are: (1) investing at all levels; (2) improving function and innovation of information technology; (3) increasing participation and feedback; and (4) creating more responsive policy. As BHC concludes activities in Da Nang, local university students will be trained on systems mapping techniques to embed systems thinking skills into the next generation of workforce, and a set of recommendations will be developed to share with the government to act on these findings.

## Introduction

Over the past few decades, Da Nang City, Vietnam, has undergone rapid growth. Thanks to its strategic location along the East–West Economic Corridor, the city has gradually become an important driving force for economic development not only in the central region but also in the entire country. Attracting various international donors and investors, Da Nang has announced an ambitious plan to become a financial hub in its implementation of the “12^th^ Politburo’s Resolution No. 43-NQ/TW on Da Nang’s development by 2030, with a vision to 2045” [1]. In response to this growth, there is a crucial need to assess how urban living in this city may influence the health of its population.

Building Healthy Cities (BHC), funded by the United States Agency for International Development (USAID), is a 5-year learning project that began in 2017, and began supporting work in Da Nang in 2019. The goal of BHC’s work is to refocus city policies, planning, and services with a health equity lens while improving data-driven decision-making for Smart Cities.

BHC defines health by both medical and non-medical factors such as the social and physical environments in which people live, work, or play. BHC employed a systems thinking approach to working with each of its four partner cities in Asia. Specifically, the project used participatory systems mapping as a tool to clearly articulate the linkages between Smart City planning (transportation, physical environment, built environment, sanitation, education, recreation and technology) and urban population health, to engage a wide range of multi-sectoral stakeholders, and to formulate a set of priority actions. This method does not make these problems any less complex; instead, it embraces that complexity and uses it to work toward a healthier system.

Da Nang City had developed a number of plans and strategies prior to BHC’s arrival, to support and promote the city’s development toward global trends in sustainable development, most notably the Da Nang City Master Plan for 2030, with a vision to 2045, approved by the Prime Minister [2]. This document reviewed and formulated urban development programs, as well as subdivision, urban, rural, and technical infrastructure planning. Additionally, human resource development has been a priority for over a decade. In 2004, the Da Nang People’s Committee, in an effort to attract talented workers to the city, launched Project 922, which sent qualified candidates to overseas training courses through fully funded scholarships [3]. Recipients participated with a commitment to return and work in the city’s governmental agencies. Da Nang has also recognized the urgent need for environmental protection and long-term sustainable development; Da Nang issued its Environmental City Initiative in 2008 [4]. Following remarkable results achieved after 12 years of implementation, the city developed the second phase of this initiative in order to transform Da Nang into an ecological city in the face of growing concerns around natural disasters, climate change, infrastructure overload, and the burden of social and health issues. One challenge the city had not yet addressed was data scarcity and community participation, especially within vulnerable groups. [1] Building on this overall success, the Da Nang city government turned its attention to increasing tourism, and recognized the need to address food safety and waste management concerns as part of that initiative. BHC, with its systems mapping approach, was engaged to identify key areas for change in support of this goal.

Other tools have been tested in the Vietnamese context to tackle health inequalities since the launch of the sustainable development goals. One example is the Urban Health Equity Assessment and Response Tool (Urban HEART) which was co-developed by the World Health Organization, city and national policy-makers, and academics and researchers in 2010 and focuses on the social, economic, and physical environment determinants of health [5]. Urban HEART “guides users through a process of bringing different stakeholders together, collecting and collectively analyzing disaggregated data on health and its determinants, and planning action to reduce inequities informed by best practices” [5]. Ho Chi Minh City is the only city in Vietnam, and one of 15 cities worldwide, piloting Urban HEART [6]. According to a study by Prasad et al., [6] all 15 pilot cities were successful in engaging multiple stakeholders; however, Ho Chi Minh City reported this process as a barrier due to a lack of skillful facilitators and expressed a need for adapting and refining some of the equity indicators. Mirzoev et al. (2019) explored the current practice of urban health planning in Hanoi and noted barriers to city planners effectively addressing urban health issues, but did not posit any new framework to overcome those issues [7]. Recent work related to urban equity identified key domains for systems strengthening, but primarily focused on informing theory [8]. Systems thinking has been tried in some small settings in Vietnam, for instance, in research by Vi Nguyen et al. (2014) which adapted the scenario planning tool as a methodological consideration to understand sanitation issues [9], but has not been implemented in a participatory or applied way as in BHC’s approach. This paper will summarize how this participatory systems mapping process was tailored to the Da Nang context, and discuss the outcomes from this work.

## Methods

Participatory systems mapping has been seen as a strong tool to bring stakeholders together in an inclusive exercise related to a specific policy, program, or urban planning initative [10]. Participatory systems mapping provides an understanding of the perspective of various stakeholders, and empowers them to produce feasible and viable solutions [11]. This particular version of applied systems mapping was designed by Engaging Inquiry (EI) and adapted for BHC. A full description of the methodology is provided in another article in this issue, Pomeroy-Stevens et al. [12]. The approach was adopted to build understanding and transparency around systemic patterns influencing current outcomes experienced in Da Nang, and to propose interventions that support the goal of becoming a healthier city.

While participatory systems mapping can be based on a combination of qualitative and quantitative data, this study relied on qualitative methods for data collection, and the analysis is evolved from grounded theory. Primary data were collected from focus group discussions, key informant interviews, and direct observation that occurred in conjunction with learning sessions and stakeholder engagement workshops to support building and visualizing causal loop diagrams, which was the primary method of synthesizing the results.

Sampling of participants was done purposively to achieve representation from all key sectors participating in BHC. These sectors were defined very clearly in a detailed memorandum of understanding signed with the city government at the start of the project, and included the Department of Information and Communication, Department of Education and Training, Department of Tourism, Department of Natural Resources and the Environment, and the Food Safety Management Authority [13]. Key disadvantaged community groups were also included in the mapping process, and these were defined during secondary data analysis and in discussion with city departments. The research methodological framework is summarized in Table 1, which also provides the total participants for each phase.

**Table 1:** Research Methodological Framework

Phase	Data Collection	Participants	Data Analysis
Launch June - July 2019	Form a research team (RT).	BHC staff, EI consultants, and city leaders.	
	Meet city government and receive approval.		
	Apply and obtain research ethics approval (exempt).		
	Conduct a city literature review and discuss between RT and city government staff to identify vision statement and framing question.		
Gain Clarity July 2019 - July 2020	Organize 4 workshops to explore 12 key forces through cause-and-effect analysis.	Da Nang governmental departments, nongovernmental organizations, universities, and related projects (*n* = 30)	Cause-and-effect analysis to develop casual loops.
	Review secondary data (reports related to Da Nang City collected and updated by the RT).		Build a Context Map on Kumu.
	Organize 3 community town halls with 11 focus group discussions (FGDs): (1) to share and refine the provisional map; and (2) gather narratives from diverse perspectives to extend relevance and depth.	Coastal community (*n* = 15)	Map feedback.
		Peri-urban community (*n* = 16)	Narrative data for each loop.
		Urban community (*n* = 14)	Finalize map.
Find Leverage October - December 2020	Target workshop participants based on expertise, sector/department and availability.	Governmental departments (health, environment, food safety), civil society organizations, and community representatives (*n* = 14)	Workshop data synthesis.
	Organize a Leverage Workshop with 4 FGDs.		Develop systemic change hypothesis/leverage opportunities.
			Visualize on Leverage Map.
Act Strategically January - October 2021	Conduct Waste Free School model pilot activity.	Participants from governmental departments (health, education, environment, food safety, information and communication), civil society organizations, private sector, researchers and community representatives (*n* = 20)	Code qualitative analysis (Nvivo) to identify key themes proposed by the participants.
	Organize online Theory of Action Workshop with 3 FGDs.		
	Review the process of building maps and propose/design solutions toward a healthier city.		

Further details about the qualitative data analysis (conducted in Word and Excel) and visualization (conducted in Kumu) can be found in Pomeroy-Stevens et al. [12]. A unique aspect of the final maps is that they are available online via Kumu for anyone to view and navigate. While the software is not entirely free to the map developer (BHC does pay a workspace fee for each city map), the access to the final maps is free and openly available. Readers will find links to the Da Nang maps throughout this article, and are encouraged to review them for more detail.

## Results

### Identifying a Vision Statement

The review of socio-economic development reports, strategies, policies, and master plans of Da Nang City allowed the authors to have a joint discussion with the city leaders to determine the following vision statement: “Da Nang is a smart, livable, sustainable city with a citizen-centric strategy delivering high quality of life and sustainable environment for citizens, while ensuring economic growth and competitiveness.” This vision statement was used to orient various focus groups as they explored the challenges Da Nang City faces as it works toward its ambitious development goals, and guided the creation of solutions for a smart, livable, and sustainable city. The question “What accounts for the current quality of citizen and environmental health in Da Nang today?” framed the inquiry and guided stakeholders to understand the current state of the system.

### Context Map Highlights and Implications

Focus group discussions resulted in a wealth of information related to development challenges and opportunities in Da Nang. Key concepts and causal relationships were synthesized into a Deep Structure loop that formed the center of the systems Context Map (https://embed.kumu.io/edc514234f111b67ae71b879f694e89e), and 10 additional loops (which expanded to 14 after community socialization), depicting key patterns of system behavior. Collectively, these loops (which can be explored in more detail in the interactive map link provided above) provide insight into four main, interconnected areas: management quality, vision and leadership, workforce capacity, and community engagement. Management quality is illustrated in the map through Da Nang’s effective management practices and policy failures, which require improved quality of information technology and data to resolve. Vision/leadership and workforce capacity are closely linked in the map; as a result of impressive leadership since the 2000s, a clear vision for the city’s development has attracted thousands of qualified laborers to Da Nang, and brought people together to build a sense of pride and personal responsibility. Throughout the map, community engagement is identified as needed to promote feedback and initiatives for sustainable development.

The Deep Structure loop named “We Are All Connected” summarizes stakeholder feedback that emphasized the significance of participation and collaboration in building a healthy city. When the level of participation and collaboration (within and between governmental departments, and across citizens, nongovernmental organizations and the private sector) is low in a Smart City, policies created are less reflective of the actual needs and opportunities present in the city, and are therefore less effective. When policies are not responsive and not able to demonstrate value, people lose faith in the government and its ability to act on behalf of its citizens. When faith in government is low, people are less likely to invest their time, energy, and resources in supporting city programs and policies, further decreasing the level of participation and the drive to collaborate that is necessary for a healthy and thriving city. During group discussions, participants shared examples of challenges faced, as well as bright spots related to this pattern.

One example of a bright spot given by stakeholders during the context mapping stage was a retirement club, named Thai Phien, which engaged retirees in providing input on city efforts. Various initiatives and policies recommended by this group have been applied (i.e. urbanization of Son Tra district, land reform, and infrastructure enforcement). People felt very proud of their contribution to this effort and were willing to do their part for it to succeed. Such policies have increased people’s trust in the government and encouraged people (both within and outside the city) to invest in Da Nang over the last two decades.

Stakeholders also identified challenging examples, for instance, the poor engagement of young families/people, particularly immigrants, in residential area meetings. As a result, they were less likely to participate in community activities or give feedback to the ward/group to make them more relevant or valuable. An example of this was a proposed program to support solid waste segregation. A women’s group proposed collecting plastic bottles and other recyclables to sell for funds to support social programs. This activity was not understood by younger generations, so they did not participate. Social divides between generations limited the potential of positive change through ward/group activities.

Looking across the system, opportunities and obstacles to healthy development in Da Nang City were identified, and are described in Table 2.

**Table 2:** Obstacles and opportunities

Obstacles	Opportunities
Limited digital data and accessibility	Strong leadership
Quality of labor force	Commitment and practices for bottom-up decision-making
Gaps between social groups	Natural resources
Lack of collaboration among stakeholders	Hospitality of city’s citizens
Limited citizen consciousness in building a healthy and smart city	Improvements of infrastructure
Hygiene issues	Utilization of smart technology for collecting data and monitoring community needs
Gaps in living conditions among different districts	International support and cooperation
Threats of environmental pollution	
Overdevelopment of tourism	

Stakeholder discussions showed confidence that Da Nang possessed the necessary conditions (human and natural resources) to promote socio-economic growth, yet there remain several obstacles. Abundant narratives reflected the necessity of a high-quality database empowering policymakers, investors, and other stakeholders to make good decisions and solve problems systematically. A mixed-methods approach was suggested to collect feedback and promote engagement from diverse population groups: a hotline (1088), a Facebook fan page, and a mobile application (Kuuho). In addition, participants emphasized the need to prioritize the engagement of low-income groups and those living with disabilities in city activities and resources to ensure that no one is left behind, thereby undermining the health of the overall system.

### Leverage Opportunities

After walking through key insights from the Context Map, leverage workshop participants formed three small discussion groups to identify and record the energy and assets already present in the system. These data provided indicators of leverage potential. Despite unique perspectives and reasoning, all three discussion groups agreed that the level of participation and collaboration, quality of information technology and data, and the pride in the city brand all held promise for creating “ripples” of positive change in the larger system. These responses were synthesized into four leverage opportunities as shown in Table 3, each of which includes the assets in the system being built upon (indicators of leverage) as well as key principles for successful action in the system.

**Table 3:** Leverage opportunities

Leverage opportunity	Description
Opportunity 1: investment at all levels	The opportunity to invest and be a part of a thriving, healthy city is extended at all levels (from external investors to city departments to citizens) through strong, inclusive leadership that encourages creativity and long-term buy in. This increased investment will in turn contribute to the quality of data available for city planning as well as the engagement of citizens and workforce in city improvement efforts.
Opportunity 2: function and innovation in information technology	Balance is created between the excitement and opportunity of new technology, and the accessibility and functionality of known and tested practices. Finding this balance will help attract and retain a skilled workforce capable of producing high-quality data for strong proactive decision-making that will support that growth through stability.
Opportunity 3: participation and feedback	Participation and feedback is prerequisite to the success of any implementation, from a sectoral program to a citywide policy. When effective management practices are employed to foster increased participation, not only in the programs themselves but also in how they are designed and improved, the understanding and awareness of city initiatives are similarly enhanced. This creates a positive feedback loop, continuing to build participation, awareness, and impact.
Opportunity 4: responsive policy	When policies are responsive and designed to best fit the current context, challenges with implementation and enforcement will be reduced. This will allow policies to operate as intended, preventing risks to health and protecting the people and environment that make the city so special.

From the leverage analysis, guiding policies were identified to support whole-system strengthening toward a healthier, resilient city. These leverage opportunities are built over the Context Map to show pathways for change. An interactive version of the Leverage Map is available here (https://embed.kumu.io/95292230cbc6ee72a89e823af965903e), and allows for readers to zoom in and see more of the detail for each leverage opportunity.

### Action Planning

City officials did not specifically ask BHC for an action plan, but given the momentum of the systems mapping process, BHC was able to hold a series of action consultations virtually (due to COVID-19) in 2021. While this list of suggested actions to move Da Nang toward its vision statement is not exhaustive, it represents a synthesis of stakeholder responses from this process.

1. Develop mobile applications to support utilization and improvement of clean/green initiatives
  There were diverse applications proposed by the participants such as providing urban services (e-parking, recyclable material collection), increasing green space, developing the circular economy, empowering start-ups, etc. Artificial intelligence-based applications could be especially adept at creating more connections among the city stakeholders (e.g. within governmental sectors, between local authorities and the private sector, and between local authorities and communities).
2. Pursue innovative approaches to sustainable development through public private partnerships
  Da Nang is a developing city with a young population, and must therefore utilize different resources to sustainably boost its growth. Public private partnerships, for instance, are a suitable model to ensure environmental health (solid waste collection, water supply in rural and remote areas) and food safety (food supply chain tracking). The government should indicate its long-term commitment to sustainable development by enacting laws and policies that make it clear to organizations and individuals that investing their time and resources will be worthwhile.
3. Ensure the accessibility of engagement for special groups in all initiatives
  As a healthy city, Da Nang must consider the full spectrum of people who live there. This is particularly important for members of the population who experience barriers to participation in services or advocacy efforts, such as low-income families, people with disabilities, people with limited access to education, and children. Thoughtful adaptations to programs or communications to increase accessibility and mitigate social gaps will promote the participation and feedback of all citizens in policy implementation.
4. Leverage the power of youth to improve community health
  Schools are an ideal setting to teach lifelong healthy behaviors. Many actions and system change efforts that benefit health and the environment are initiated by youth action. Working with students, teachers, and the community on easy-to-adopt habits of recycling, composting, and environmental protection begins a cycle of environmental awareness during a child’s formative years. The ripple effect of these actions impacts their families and surrounding communities. Empowering and engaging students inspire them to become lifelong stewards of their environment and give them a voice in community decision-making.

## Discussion

Researchers worldwide have developed and advanced various methods, tools, and approaches to understand urban system complexity [14]. A healthy city requires collective leadership [15], accessible data, [8] and multi-stakeholder engagement [6]. However, there is often a lack of collaboration in urban planning and development between governments and other stakeholders. For instance, a 2021 baseline study on local civil society organizations working in biodiversity conservation and environmental sustainability in Central Region and Central Highlands of Vietnam reported an essential need to promote and strengthen partnerships between nongovernmental organizations and the local governments [16]. In two other examples from Vietnam, although the most recent urban planning procedures require community consultation, this consultation process has not been taken seriously and most of the decisions have been made solely by government agencies, which has led to inapplicable and impractical policies [17, 18]. Therefore, participatory design in urban planning is crucial to ensure that policies are relevant and bridge the policy-implementation gap [19, 20].

BHC’s participatory systems mapping approach was applied to understand the current health of Da Nang City. The process included similar steps as suggested by the Urban HEART tool [21] such as building an inclusive team (BHC engaged 108 multi-sectoral stakeholders from governmental departments, civil society organizations, schools, communities, private sector, and research institutions), assembling relevant and valid data, generating evidence, and identifying the best response for future interventions.

However, BHC’s approach is unique in that it provided opportunities for all stakeholders of Da Nang to participate side-by-side and uncover key patterns driving outcomes experienced across the city. The participatory systems mapping approach facilitates deep analysis to comprehend the underlying system structures and the feedback mechanisms that influence the system over time [22]. In this Da Nang example, the Context Map provides a comprehensive picture of the city in a way that is dynamic, aligns stories from diverse viewpoints, and provides clear evidence of change potential. Across the arc of the project, important recommendations for policy development or improvement, and numerous initiatives were shared between residents, managers, and officials through the workshops. The result was increased understanding and consensus in policy implementation, across sectors and levels of influence. Notably, the project partnered with the University of Da Nang, a regional university with national prestige and strong influence on society, to share the research results with the city government.

Additionally, by focusing on systemic patterns, challenges were contextualized as the result of interconnected structures, behaviors, and mental models. In this way, the perception of judgement or blame was removed and key actors were more willing to come together to understand the shared opportunity for positive change. For contexts such as Da Nang where speech or activity that is seen as critical to the government is met with resistance, this approach supports open conversation in a safe way that is grounded in the assumption that it is systemic forces that are perpetuating difficult outcomes that nobody really wants.

Recognizing that teachers and students are important change agents of community engagement [23, 24], BHC facilitated a partnership between the food safety and solid waste management sectors to develop activities in schools [25, 26]. By building on the energy, enthusiasm, and potential already present in communities around the school environment, progress on implementation goals was smooth and had greater impact. Despite the COVID-19 pandemic, hands-on trainings were conducted using animated videos, and composting programs and innovative trash bins were established at schools.

The project faced certain limitations, including that the qualitative data collection process used to build the maps was completed in Vietnamese and then translated to English. Although the primary members of the research team were native Vietnamese and fluent English speakers, some nuanced detail and meaning may have been lost. Additionally, in group discussions, some participating representatives of city government agencies were hesitant to share their opinions and information on “sensitive” issues, specifically any inadequacy of policies or policy operations. Finally, due to safety measures necessary during a global pandemic, the Theory of Action workshop was conducted online, resulting in less effective data collection than expected.

Lessons learned from the initial success of the BHC project in Da Nang may be applied to other cities in Vietnam, and beyond. First, the facilitation of robust system learning and intervention design requires the following: (1) selection of enthusiastic and collaborative participants; (2) agreement of leaders of the local organizations to participate in the project workshops, or nominate best-fit representation; (3) clear explanation of the process and objectives in each engagement; and (4) talented and experienced facilitators and coordinators who can foster genuine engagement from all participants. Second, workshop participants not only supported project goals but also developed insights into the concept of systems thinking and the multi-perspective approach, as well as actionable tools they could bring back to their own project or organization. Through this experience, they had the opportunity to broaden their horizons on various aspects of urban management and widen their network beyond typical interactions to include new governmental organizations, private sector actors, and local nongovernmental organizations. Third, the partnership between BHC and the University of Da Nang strongly benefitted the overall success of this project. This collaboration enabled the uptake of systems mapping tools to promote capacity building, and sustain and extend the project results for further research.

## Conclusion

Systems mapping provided an opportunity to support Da Nang’s ongoing and ever-evolving journey of building a healthy city. The research results captured the city’s current status in its development trajectory and movement toward its vision as a smart, healthy, and livable city. Overall, Da Nang has many bright spots that already support a high quality of life and culture for its citizens, such as a strong workforce, good leadership, and renowned hospitality. The resulting Context Map’s Deep Structure and other causal loops highlight the significance of participation and collaboration among multi-sector stakeholders to mobilize internal and external investment at all levels to make Da Nang a thriving and healthy city.

## Data Availability

The resulting Context (https://embed.kumu.io/edc514234f111b67ae71b879f694e89e) and Leverage Maps (https://embed.kumu.io/95292230cbc6ee72a89e823af965903e) are available on the platform Kumu.
